# Validation of a guidelines-based digital tool to assess the need for germline cancer genetic testing

**DOI:** 10.1186/s13053-024-00298-0

**Published:** 2024-11-08

**Authors:** Callan D. Russell, Ashley V. Daley, Durand R. Van Arnem, Andi V. Hila, Kiley J. Johnson, Jill N. Davies, Hanah S. Cytron, Kaylene J. Ready, Cary M. Armstrong, Mark E. Sylvester, Colleen A. Caleshu

**Affiliations:** 1 Genome Medical, 611 Gateway Blvd Suite 120, South San Francisco, CA 94080 USA; 2 Northside Hospital, 1000 Johnson Ferry Rd NE, Atlanta, GA 30342 USA; 3 GeneMatters, 611 Gateway Blvd Suite 120, South San Francisco, CA 94080 USA

**Keywords:** Hereditary cancer, Genetic testing, Cancer risk assessment

## Abstract

**Background:**

Efficient and scalable solutions are needed to identify patients who qualify for germline cancer genetic testing. We evaluated the clinical validity of a brief, patient-administered hereditary cancer risk assessment digital tool programmed to assess if patients meet criteria for germline genetic testing, based on personal and family history, and in line with national guidelines.

**Methods:**

We applied the tool to cases seen in a nationwide telehealth genetic counseling practice. Validity of the tool was evaluated by comparing the tool’s assessment to that of the genetic counselor who saw the patient. Patients’ histories were extracted from genetic counselor-collected pedigrees and input into the tool by the research team to model how a patient would complete the tool. We also validated the tool’s assessment of which specific aspects of the personal and family history met criteria for genetic testing. Descriptive statistics were used.

**Results:**

Of the 152 cases (80% female, mean age 52.3), 56% had a personal history of cancer and 66% met genetic testing criteria. The tool and genetic counselor agreed in 96% of cases. Most disagreements (4/6; 67%) occurred because the genetic counselor’s assessment relied on details the tool was not programmed to collect since patients typically don’t have access to the relevant information (pathology details, risk models). We also found complete agreement between the tool and research team on which specific aspects of the patient’s history met criteria for genetic testing.

**Conclusion:**

We observed a high level of agreement with genetic counselor assessments, affirming the tool’s clinical validity in identifying individuals for hereditary cancer predisposition testing and its potential for increasing access to hereditary cancer risk assessment.

## Background

At least 5–10% of cancer diagnoses have a hereditary basis, arising from a germline pathogenic variant in a cancer predisposition gene [1]. Within the general population, 8% of individuals are estimated to have a likely pathogenic or pathogenic variant in these genes, yet the vast majority of these individuals do not know they possess this risk [2–5]. Identification of individuals with a hereditary risk of cancer allows for personalized care such as more frequent and earlier cancer screening, as well as risk-reducing surgeries. These measures have been shown to lead to earlier cancer diagnoses, improved prognosis, and/or prevention of cancer [6–8]. In addition, among patients with cancer, identification of individuals with certain germline pathogenic variants is necessary for personalized cancer treatment such as PARP inhibitors for those with *BRCA1/2* likely pathogenic or pathogenic variants and immune checkpoint therapies for those with Lynch syndrome [9, 10]. Given these clinical benefits, multiple professional guidelines recommend that oncologists, obstetricians-gynecologists, and primary care physicians perform hereditary cancer risk assessment [1, 6, 11–13].

There is ample evidence that in both non-specialty and oncology settings, hereditary cancer risk assessment is not performed as recommended by guidelines [2, 4, 14–17]. In one study, fewer than 20% of women with a personal and/or family history of breast or ovarian cancer who meet National Comprehensive Cancer Network (NCCN) criteria for germline cancer genetic testing have had such testing [2]. Most individuals who haven’t had testing reported never discussing testing with a healthcare provider [2]. In a 2022 study of over 279,000 women receiving primary care at the Cleveland Clinic, only 22% of high-risk women had been referred for genetic testing [4]. Additionally, that study found disparities in referrals based on race, with Black individuals significantly less likely to be referred than White individuals. A 2023 study of over 1.3 million cancer patients found that while rates of germline genetic testing after a cancer diagnosis have increased over time, such testing remains heavily underutilized [16].

Investigations into the reasons that providers do not perform guideline-recommended hereditary cancer risk assessments have revealed that providers perceive such assessments as valuable and important, but they face many barriers to performing them for their patients [18–21]. Non-genetics providers feel they lack sufficient genetics expertise, do not feel confident answering patient questions related to genetic risk and genetic testing, and have difficulty staying up to date with advances in genetic testing [18–21]. In addition, providers report they do not have time to adequately assess and counsel patients about hereditary cancer risk [18, 21]. This is understandable, given it can take up to 30 min to collect the extensive family history that is often needed to determine if a patient meets guideline-based criteria [22]. Furthermore, such criteria are complex and frequently change, making it difficult for providers to apply them. New approaches to hereditary cancer risk assessment are needed that address these barriers. Several paper-based screening tools have been developed; these are often brief forms completed by patients and scored by clinic staff. While they increase the identification of patients at high risk, they only cover a small subset of testing criteria and thus miss many patients who qualify for genetic testing [23–26].

Digital tools have the potential to cover far more testing criteria and to assess patients in an automated fashion that does not depend on clinic staff. A variety of such solutions have arisen in recent years [27–31]. This includes automated algorithms that leverage family history information already captured in the electronic health record [31] as well as patient-facing digital tools that perform hereditary cancer risk assessment based on patient-entered personal and family history [27, 28, 30]. Studies have found that such digital tools effectively identify at-risk patients who would have been overlooked [25, 27, 30, 32–35]. Importantly, patients report high levels of satisfaction with digital tools in genomics care [20, 27, 33, 36–38]. While these studies suggest digital tools may increase access to genetics care, further research is needed to ensure that is the case and to assess any ways in which they act as a barrier.

**Fig. 1:**
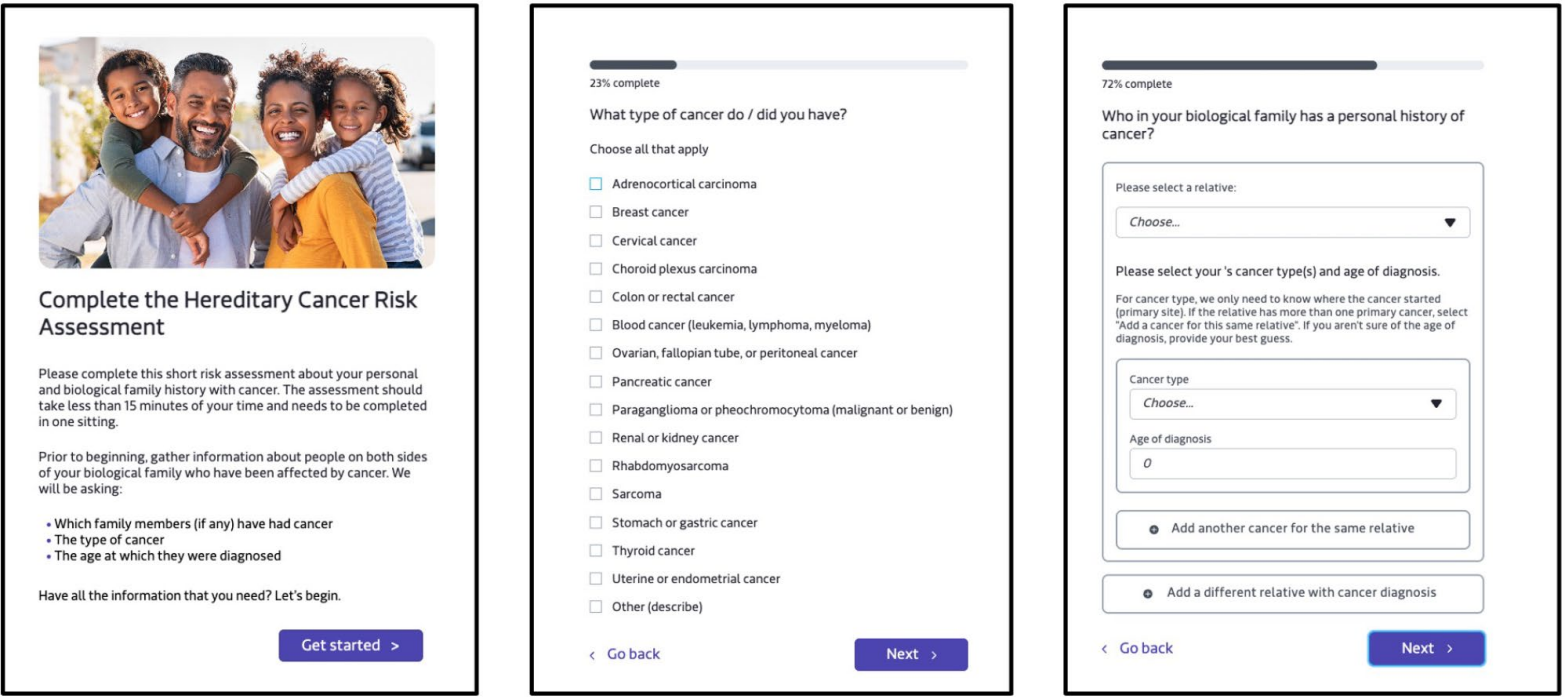
**The hereditary cancer risk assessment digital tool (RISE)**. Screenshots of various steps in the digital tool including instructions to the patient, sample personal history questions, sample family history questions. Images courtesy of Genome Medical. Used with permission

We developed RISE Risk Assessment Module: Hereditary Cancer to help providers perform hereditary cancer risk assessment without a significant time or process burden for them or their clinic staff (Fig. 1). This is a brief, patient-administered web-based tool designed to assess whether germline cancer genetic testing may be indicated, consistent with multiple national guidelines. A typical workflow involves the patient completing the tool in advance of an appointment or in the waiting room. The assessment then is displayed to the patient directly in the tool and available to the provider via PDF [35]. The tool is available for use in the United States via licensing from Genome Medical, a private genetics services company. It was created by a team at Genome Medical that includes genetic counselors (GCs) with expertise in oncology, product managers, user experience designers, and software engineers. Patients answer questions about their personal and family history and the algorithm underlying the tool assesses whether that history indicates genetic testing is appropriate (Fig. 1). The algorithm includes 98 discrete rules that each recognize one or more aspects of personal and/or family history that meet criteria for genetic testing. Triggering of one or more rules leads to an assessment of meeting criteria. An example of a rule is having a first-degree relative with prostate cancer and two other close relatives with prostate or breast cancer. To increase usability and efficiency, the history questions are programmed with skip logic so patients only see questions relevant to them. The tool includes 3 demographic questions. The number of personal history and family history questions depends on the extent of those histories; a patient with multiple cancer diagnoses will answer more questions than one who has not been diagnosed with cancer. The history collection is modeled after the details that would be collected during a 3-generation pedigree during a cancer genetic counseling consultation. Sample questions are displayed in Fig. 1. When patients report a personal or family history of a cancer diagnosis, the tool asks the patient to document the specific type of cancer (i.e., breast, prostate, etc.), relative diagnosed (i.e., mother, sister, niece, etc.), and age of onset. Questions also address prior genetic testing in the family. Hereditary cancer risk assessment tools that have been studied to date assess for increased risk of one or a combination of hereditary breast and ovarian cancer, Lynch syndrome, and polyposis syndromes [23, 25, 27–30]. The tool under study here was programmed to detect hereditary risk for a wider range of cancers, hereditary cancer syndromes, and tumors (Table 1). The algorithm is updated as guideline updates are released. The platform is HIPAA-compliant and Service Organization Control Type 2 (SOC2) certified.

**Table 1:** RISE assesses hereditary risk for a variety of cancers, tumors, and hereditary cancer syndromes

Cancers/tumors	Hereditary cancer syndromes
Breast	Attenuated familial adenomatous polyposis (AFAP)
Colorectal	Classical familial adenomatous polyposis (FAP)
Gastric	Cowden
Kidney	Hereditary breast and ovarian cancer (HBOC)
Ovarian	Lynch
Paraganglioma	Multiple endocrine neoplasia type 2 (MEN2)
Pheochromocytoma	MUTYH-associated polyposis (MAP)
Prostate	Von Hippel–Lindau (VHL)
Uterine	

This is a non-exhaustive list of the tumors, cancers, and hereditary cancer syndromes for which RISE’s algorithm assesses risk

While risk assessment digital tools like this one may increase access to genetic testing, appropriate validation of such tools must be performed to ensure the accuracy of their assessments [20]. Of those that do report validation, there are mixed results on the sensitivity and specificity of tools. One such study showed 100% sensitivity and 99.5% specificity, however, the low-risk cases were fabricated and the validation only covered assessment for hereditary breast-ovarian cancer and Lynch syndrome [28]. Validation of another digital risk assessment tool found it failed to identify half of the individuals that genetics clinicians assessed to be at an increased risk for a hereditary cancer predisposition [30].

We sought to validate RISE Risk Assessment Module: Hereditary Cancer against assessments made by GCs specialized in oncology.

### Methods

To assess the clinical validity of the tool, the tool’s assessment of the patient meeting criteria for genetic testing was compared to the assessment made by the board-certified cancer GC who previously saw the patient for clinical care. The tool’s assessment was performed retrospectively and as part of this study only (not part of the patient’s clinical care).

### Patients

Cases were drawn from patients seen for pre-test genetic counseling for hereditary cancer risk in Genome Medical’s genetic counseling practice between July 23, 2020, and October 23, 2020. Genome Medical is a private for-profit entity that provides telehealth genetic counseling services across the United States and Canada. We used purposive sampling to select cases that met criteria for genetic testing (as assessed by the GC who saw the patient for clinical care) to ensure that the sample covered the criteria most frequently invoked in clinical practice and for variance in cancer types. Cases that did not meet criteria for genetic testing (as assessed by the GC who saw the patient for clinical care) were consecutively selected. Purposive sampling methods were not used for these cases because cases not meeting criteria cannot be sampled based on specific rules in the same way that cases meeting criteria can be.

### Data collection

Patient demographics (sex, race, ethnicity, ancestry), personal history of polyps or cancer, family history of cancer, and the GC’s assessment of whether the patient met criteria were extracted via a retrospective review of the electronic medical record. The GC’s three-generation pedigree was reviewed to extract the patient’s family history of cancer, which was then entered into the tool so as to mimic how a patient would complete the tool. The tool’s assessment was then collected.

### Validation

Validity was operationalized as the proportion of cases where the tool’s assessment of whether the patient met criteria for genetic testing agreed with the assessment made by the GC who saw the patient clinically. When the tool’s assessment and the assessment made by the GC disagreed, a senior cancer GC (AD) reviewed the case in detail to determine the origin of the disagreement.

Performance of the tool depends on the accuracy of the underlying algorithm in assessing that specific aspects of the patient’s personal and/or family history meet criteria. To further evaluate the performance of the tool, we compared the tool’s and research team’s assessment of which specific aspects of the patient’s personal and/or family history met criteria. This involved comparing the personal and/or family history-based rule(s) in the tool’s algorithm that were triggered for a given case to the research team’s separate and independent assessment of which personal and/or family history-based rule(s) should have been triggered. This analysis was aimed at evaluating the rules that come up most frequently in clinical practice. As such, only a subset of cases were analyzed, enough to evaluate all high-frequency rules. The frequency of rules was rated by a senior cancer GC (AD).

## Results

The dataset consisted of 152 patients seen for pre-test cancer genetic counseling with two-thirds meeting criteria for genetic testing (per GC assessment). Half had a personal history of cancer, with a variety of cancer types represented (Table 2).

**Table 2:** Patient characteristics

		Met criteria^a^	
	All cases	Yes	No
**Age (mean [standard deviation])**	52.3 (SD 15.9)	55.3 (SD 15.4)	45.8 (SD 15.3)
**Sex**			
Female	121/152 (79.6%)	78/99 (78.8%)	40/47 (85.1%)
Male	31/152 (20.4%)	21/99 (21.2%)	7/47 (14.9%)
**Race**			
American Indian/Alaska Native	1/138 (0.7%)	1/98 (1.0%)	-
Asian	3/138 (2.2%)	1/98 (1.0%)	2/40 (5.0%)
Black or African American	11/138 (8.0%)	8/98 (8.2%)	3/40 (7.5%)
Other Race	7/138 (5.1%)	5/98 (5.1%)	2/40 (5.0%)
White	116/138 (84.1%)	83/98 (84.7%)	33/40 (82.5%)
**Ethnicity**			
Hispanic or Latino	12/134 (9.0%)	6/91 (6.7%)	6/43 (14.0%)
**Personal History of Cancer**			
Any diagnosis of cancer	85/152 (55.9%)	69/99 (69.7%)	16/47 (34.0%)
Multiple primary diagnoses	27/152 (17.8%)	24/99 (24.2%)	3/47 (6.4%)
*Cancer type*			
Breast	30/152 (19.7%)	21/99 (21.2%)	9/47 (19.1%)
Colorectal	15/152 (9.9%)	14/99 (14.1%)	2/47 (4.3%)
Uterine	14/152 (9.2%)	14/99 (14.1%)	-
Prostate	10/152 (6.6%)	10/99 (10.1%)	-
Ovarian	7/152 (4.6%)	7/99 (7.1%)	-
Pancreatic	7/152 (4.6%)	7/99 (7.1%)	-
Kidney	2/152 (1.3%)	1/99 (1.0%)	1/47 (2.1%)
**Family History of Cancer** ^b^	126/152 (82.9%)	91/99 (91.0%)	35/47 (74.5%)
*By cancer type* ^c^			
Breast	86/152 (56.6%)	66/99 (66.7%)	20/47 (42.6%)
Prostate	43/152 (28.3%)	36/99 (36.4%)	7/47 (14.9%)
Colorectal	42/152 (27.6%)	31/99 (31.3%)	11/47 (23.4%)
Pancreatic	33/152 (21.7%)	22/99 (22.2%)	11/47 (23.4%)
Ovarian	23/152 (15.1%)	21/99 (21.2%)	2/47 (4.3%)
Uterine	14/152 (9.2%)	12/99 (12.1%)	2/47 (4.3%)
Kidney	13/152 (8.6%)	10/99 (10.1%)	3/47 (6.4%)
Stomach or Gastric	11/152 (7.2%)	9/99 (9.1%)	2/47 (4.3%)
Thyroid	8/152 (5.3%)	6/99 (6.1%)	2/47 (4.3%)

SD = standard deviation

^a^ Met criteria based on genetic counselor’s assessment

^b^Number of cases in which the patient reported at least one family member with breast, prostate, colorectal, pancreatic, ovarian, uterine, kidney, stomach/gastric, and/or thyroid cancer. Family history was extracted from 3-generation pedigrees collected by the genetic counselor who saw the patient for clinical care

^c^ Total number of patients with at least one family member with a cancer diagnosis of that cancer type

**Fig. 2:**
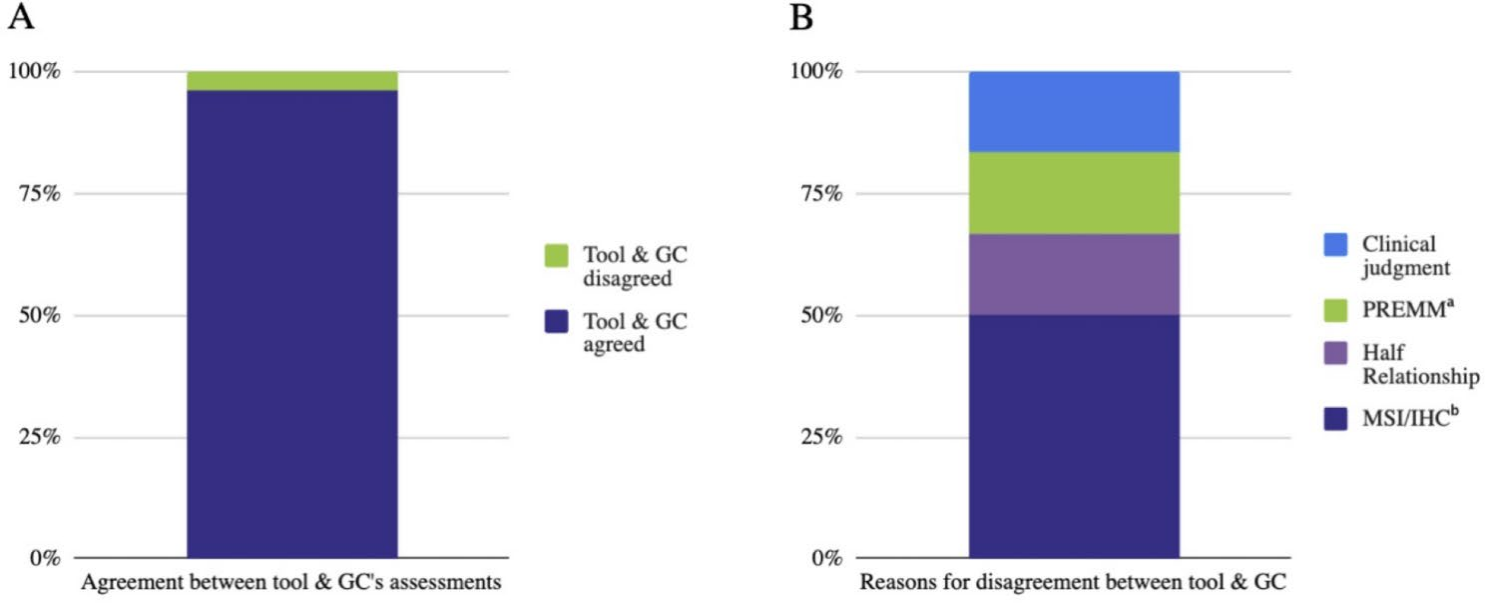
**Level of agreement between tool and genetic counselor** (**A**) Stacked bar chart showing the percentage of cases with agreement in assessments made by the tool and the GC (96% [146/152]) (**B**) Reasons for disagreement in the 3.9% (6/152) of cases where the tool’s and GC’s assessments differed. The tool does not ask about clinical details patients typically cannot report such as MSI/IHC and risk of having a germline pathogenic variant based on the PREdiction Model for gene Mutations (PREMM5), nor does it account for half relationships. In one case the GC applied their clinical judgment in interpreting a patient’s history of polyps in a manner that the tool could not. (**a**) PREMM = PREdiction Model for gene Mutations. (**b**) MSI/IHC = Microsatellite Instability/Immunohistochemistry

In 96% (146/152) of cases, the tool’s assessment of whether the patient met criteria for genetic testing agreed with the GC’s assessment (Fig. 2). Among patients who met criteria (by GC assessment), there was 95% (94/99) agreement between the tool and GC. Among patients who did not meet criteria (by GC assessment), there was 98% (46/47) agreement between tool and GC.

For the cases (3.9% [6/152]) where there was disagreement between the tool and GC, we examined why the assessments differed (Table 3). Of note, no disagreements in assessment occurred because of a rule in the tool’s algorithm not being triggered when it should have been. Most differences in assessment (67%) occurred because the GC assessment depended on a specific aspect of history that the tool did not ask about. This included microsatellite instability/immunohistochemistry (MSI/IHC) results (50%) and risk of having a germline pathogenic variant based on the PREdiction Model for gene Mutations (PREMM5; 16.7%) [39]. Disagreement occurred in one case because the GC applied their clinical expertise in interpreting the patient’s polyp history as meeting criteria based on likely polyp type, while the tool assessed the patient as not meeting criteria because polyp type was unknown (16.7%). The final case of disagreement arose because the tool does not currently allow entry of half-relationships (16.7%).

**Table 3:** Reasons for genetic counselor and tool disagreement

Case(s)	Meets criteria?		Reason for Disagreement
	Tool	GC	
1, 2, 3	No	Yes	Patient met criteria solely based on MSI/IHC results which the genetic counselor had access to but was an aspect of history that was intentionally not included in the tool since patients usually cannot self-report this
4	No	Yes	Patient met criteria solely based on having an increased risk of having an MMR gene pathogenic variant based on predictive models (PREMM5) which the genetic counselor had access to but was an aspect of history that was intentionally not included in the tool since patients usually cannot self-report this
5	No	Yes	Genetic counselor used clinical judgment in interpreting the patient’s polyp history, assessing that the polyps were likely of a type that would meet criteria. The tool did not identify this patient as meeting criteria because the specific polyp type was unknown
6	Yes	No	The digital tool does not account for half relationships. The tool’s assessment resulted from counting a half sibling with cancer as a full sibling

GC: genetic counselor; MSI: Microsatellite instability; IHC: Immunohistochemistry; DNA Mismatch Repair gene (MMR gene): *MLH1*, *MSH2*, *MSH6*, and *PMS2*; PREMM5: PREdiction Model for gene Mutations

For the evaluation of the performance of individual rules, all high-frequency rules were assessed after analysis of 62 of 152 cases (Table 4). In all cases, the research team and the tool agreed on which aspects of the patient’s personal and/or family history met criteria for genetic testing. Across these 62 cases, specific aspects of the patient’s personal and/or family history were recognized by 65 different rules in the tool’s algorithm, and these rules were triggered a total of 269 times, with complete agreement between the tool and research team each time they were triggered (100% [269/269]). Each rule was triggered by a mean of 3.8 cases (SD 3.5) and the mean number of rules triggered per case was 4.1 (SD 2.7). All algorithm rules that are used in clinical practice with the highest frequency were validated individually (100% [37/37]), as were most intermediate frequency rules (68.4% [26/38]) (Table 5). The majority of individual rules in the following cancer types were validated: breast, ovarian, pancreatic (73.3% [33/45]); colorectal, endometrial (93.3% [14/15]); prostate (69.6% [16/23]) (Table 5).

**Table 4:** Characteristics of cases used for rule validation

	54.3 (SD 13.3)
**Sex**	
Female	50/62 (80.6%)
Male	12/62 (19.4%)
**Personal history of cancer**	
Any diagnosis of cancer	34/62 (54.8%)
Multiple primary diagnoses	10/62 (16.1%)
*Cancer Type*	
Breast	19/62 (30.1%)
Prostate	10/62 (16.1%)
Colorectal	5/62 (8.1%)
Uterine	4/62 (6.5%)
Kidney	2/62 (3.2%)
Ovarian	2/62 (3.2%)
Pancreatic	2/62 (3.2%)
**Family History of Cancer**	
At least one relative with cancer^a^	58/62 (93.5%)
*Cancer type* ^b^	
Breast	43/62 (69.4%)
Prostate	27/62 (43.6%)
Colorectal	20/62 (32.3%)
Ovarian	12/62 (19.4%)
Pancreatic	15/62 (24.2%)
Uterine	9/62 (14.5%)
Kidney	9/62 (14.5%)
Stomach or Gastric	6/62 (9.7%)
Thyroid	2/62 (3.2%)

^a^ Number of cases in which the patient reported at least one family member with breast, prostate, colorectal, pancreatic, ovarian, uterine, kidney, stomach/gastric, and/or thyroid cancer. Family history was extracted from 3-generation pedigrees collected by the genetic counselor who saw the patient for clinical care

^b^ Total number of patients with at least one family member with a cancer diagnosis of that cancer type

**Table 5:** Rules validated

	Frequency of rule^a^			Total
	High	Moderate	Low	
*Cancer group*				
Breast, Ovarian, Pancreatic	26/26 (100%)	7/10 (70%)	0/9 (0%)	33/45 (73%)
Prostate	5/5 (100%)	10/15 (67%)	1/3 (33%)	16/23 (70%)
Colorectal/Endometrial	5/5 (100%)	9/10 (90%)	-	14/15 (93%)
Thyroid	-	-	0/1 (0%)	0/1 (0%)
Kidney	-	0/2 (0%)	1/1 (100%)	1/3 (33%)
Gastric	-	0/1 (0%)	0/8 (0%)	0/9 (0%)
Neuroendocrine/Adrenal	-	-	0/1 (0%)	0/1 (0%)
Known Familial Likely pathogenic or pathogenic variant	1/1 (100%)	-	-	1/1 (100%)
**Total**	37/37 (100%)	26/38 (68%)	2/23 (9%)	65/98 (66%)

^a^A senior cancer genetic counselor (AD) categorized rules by how frequently they are used in clinical care

## Discussion

We observed a high level of agreement between the tool and GCs, which suggests that the tool is accurate in its assessments of whether patients meet criteria for genetic testing. The rate of agreement we observed was markedly higher than that seen by Cohn et al., similar to that seen by Baumgart et al. and slightly lower than that reported by Bucheit et al. [28, 30, 40]. It is also at the high end of the range of accuracy reported by the U.S Preventive Services Task Force (USPSTF) in their review of several less automated hereditary cancer risk assessment tools [41]. Furthermore, we found complete agreement between the tool and the study team on which specific aspects of a patient’s personal and family history met criteria. This is particularly critical when hereditary cancer risk assessment is done in primary care or other population-based settings since many unaffected patients in such settings qualify for genetic testing based on just one aspect of their family history [35]. Taken together, these findings suggest the tool has an acceptable level of accuracy that is higher than or comparable to other risk assessment tools. It is also notable that the tool was validated using cases meeting criteria for a variety of hereditary cancer predispositions. Other digital risk assessment tools, and also validation of those tools, have primarily focused only on *BRCA1/2* and Lynch syndrome [27, 28, 30]. In contrast, the current validation covered risk for a wide range of cancers, cancer syndromes, and tumors, all of which the tool is programmed to detect (Table 1). Another strength of this study is the use of real patient cases, in contrast to prior work on validation of digital risk assessment tools which has relied, at least in part, on fictitious cases [28].

It is worth considering the minority of cases where the tool and GC disagreed. Of note, none of the disagreements were due to errors in the functioning of the tool. Most disagreements occurred because of history questions that were intentionally left out of the tool (ex. MSI/IHC [3 cases], PREMM5 [1 case]) due to our clinical experience that patients do not have the necessary information to answer such questions. Asking more questions and asking questions patients can’t answer can increase cognitive burden and decrease usability, both of which have been shown to decrease patient engagement with digital health tools [42, 43]. RISE was intentionally designed to be brief to maximize completion rates; we’ve found that more than 95% of patients who start the tool complete it, with most patients completing the tool in less than 3 min [35]. Given that MSI/IHC contributed to disagreement in multiple cases, we could add a question on that to the tool and then study whether patients can answer it and whether completion rates decrease. An additional area for improvement of the tool is the addition of half-relationships, as this contributed to disagreement in one case.

The high level of agreement between the tool and GCs that we observed, combined with prior research on feasibility and acceptability of genomics digital tools [27, 33, 36–38, 44–46], supports them as promising solutions to increasing access to hereditary cancer risk assessment without burdening clinicians. In a recent systematic review, Lee et al. found that 84% of 87 studies on digital tools in genomics reported a positive outcome and that digital tools increased provider efficiency and decreased the time providers need to spend with patients [36]. Hereditary cancer risk assessment tools could also make periodic re-assessment more feasible, which is recommended by guidelines [6]. While recent studies demonstrate that such tools effectively identify at-risk patients who were otherwise un-ascertained, they also find that additional work is needed to increase the proportion of these at-risk patients who go on to have genetic testing [47, 48]. This demonstrates that innovation and practice improvement are needed at multiple steps in the care pathway to ensure access to the benefits of genomic medicine. Finally, implementation studies on digital tools in genomic medicine are needed to ensure that they can be effectively integrated into care and that they do not unintentionally increase barriers to genetic testing.

### Limitations

An important limitation of this work is that patient histories were not entered by patients themselves, but instead by the research team. While the validation covered a range of aspects of personal and family history and types of hereditary cancer risk, it was not exhaustive; we did not validate every rule in the algorithm or every way a given rule could be triggered. Additionally, since the cases included in our study had already been assessed as needing genetic counseling, they are not representative of a lower-risk population. We did not have sufficient variance in disagreement in assessment or either race or ethnicity to be able to investigate disparities in the tool’s performance. Multiple studies have found disparities in cancer genetics care based on race ethnicity, and socioeconomic status [4, 49, 50]. While digital tools have potential to reduce disparities, care in their design, implementation, and evaluation is needed to ensure they benefit patients equitably. Our sampling methods did not allow for calculation of sensitivity and specificity. This study was conducted by researchers at Genome Medical and the tool is offered commercially by Genome Medical. This may have contributed to bias, though we have made efforts to mitigate that.

## Conclusion

We observed a high degree of agreement between the digital tool and cancer GCs’ assessments of whether patients meet criteria for germline genetic testing. Combined with prior findings on feasibility, acceptability, and efficiency of digital tools in genomics, our results suggest that RISE Risk Assessment Module: Hereditary Cancer could help increase access to hereditary cancer risk assessment and genetic testing without significantly burdening clinicians.

## Data Availability

Data is available upon reasonable request made to the corresponding author.

## Abbreviations

GC: Genetic Counselor
MSI/IHC: Microsatellite instability/immunohistochemistry
PREMM5: PREdiction Model for gene Mutations
SOC2: Service Organization Control Type 2
USPSTF: U.S Preventive Services Task Force
